# Conventional partial pancreatoduodenectomy versus an extended pancreatoduodenectomy (triangle operation) for pancreatic head cancers—study protocol for the randomised controlled TRIANGLE trial

**DOI:** 10.1186/s13063-023-07337-6

**Published:** 2023-05-30

**Authors:** Patrick Heger, Thilo Hackert, Markus K. Diener, Manuel Feißt, Christina Klose, Colette Dörr-Harim, Friedhelm Möhlenbrock, Markus W. Büchler, André L. Mihaljevic

**Affiliations:** 1 Department of General and Visceral Surgery and Clinical Trial Centre of the Department of General and Visceral Surgery (ulmCARES), University Hospital Ulm, Albert-Einstein-Allee 23, 89081 Ulm, Germany; 2 Department of General, Visceral and Transplantation Surgery, University Hospital Heidelberg, Im Neuenheimer Feld 420, 69120 Heidelberg, Germany; 3 Medical Faculty, Ulm University, Meyerhofstraße M28, Ulm, 89081 Germany; 4 Department of General and Visceral Surgery, University Hospital Freiburg, Hugstetter Straße 55, 79106 Freiburg, Germany; 5 Institute of Medical Biometry (IMBI), University of Heidelberg, Im Neuenheimer Feld 130.3, 69120 Heidelberg, Germany; 6 Arbeitskreis der Pankreatektomierten e.V, Thomas-Mann-Straße 40, 53111 Bonn, Germany

**Keywords:** Pancreatic neoplasms, Carcinoma, Pancreatic ductal, Pancreaticoduodenectomy, Randomised controlled trial

## Abstract

**Background:**

Pancreatic ductal carcinoma (PDAC) is the fourth most frequent cause of cancer-related death in the Western world, and its incidence is rising. In patients that undergo curative resection, local recurrence (LR) is frequent. A recently described surgical technique of extended pancreatoduodenectomy (PD) termed the TRIANGLE operation has been proposed as a promising approach to reduce LR and improve disease-free survival in PDAC patients.

**Methods:**

The TRIANGLE trial is a multicentre confirmatory randomised controlled superiority trial with two parallel study groups. A total of 270 patients with suspected or histologically confirmed pancreatic head cancer scheduled for PD will be included in the trial and randomly assigned to the intervention group (extended PD defined as Inoue level 3 dissection along the superior mesenteric and celiac artery as well as removal of all soft tissue in the so-called triangle between the celiac artery, the SMA and the mesenterico-portal axis) or the control group (conventional PD with lymphadenectomy and removal of soft tissue according to current guidelines). The primary endpoint of the trial will be the disease-free survival of patients. Other perioperative outcomes as well as oncological parameters and patient-reported outcomes will be analysed as secondary outcomes.

**Discussion:**

Despite multimodal treatment, LR remains high and disease-free survival is limited following PD for PDAC. The TRIANGLE operation could address these shortcomings of conventional PD as indicated in several retrospective studies. However, this technique could be associated with more adverse events for patients including intractable diarrhoea. The TRIANGLE trial will close the evidence gap as well as offer a risk-benefit assessment of this more radical approach to PD.

**Trial registration:**

German Clinical Trials Register DRKS00030576 (UTN U1111-1243-4412) 19th December 2022.

**Supplementary Information:**

The online version contains supplementary material available at 10.1186/s13063-023-07337-6.

## Administrative information

Note: The numbers in curly brackets in this protocol refer to the SPIRIT checklist item numbers. The order of the items has been modified to group similar items (see http://www.equator-network.org/reporting-guidelines/spirit-2013-statement-defining-standard-protocol-items-for-clinical-trials/).

**Table:** 

Title {1}	Conventional partial pancreatoduodenectomy versus an extended pancreatoduodenectomy (triangle operation) for pancreatic head cancers—study protocol for the randomised controlled TRIANGLE trial
Trial registration {2a and 2b}	DRKS00030576 (German Clinical Trials Register)
Protocol version {3}	Version 1.0, 15 November 2022
Funding {4}	German Federal Ministry of Education and Research (Bundesministerium für Bildung und Forschung, BMBF). Funding number: 01KG2129
Author details {5a}	Patrick Heger, Colette Dörr-Harim, André L. Mihaljevic (current address) Department of General and Visceral Surgery and Clinical Trial Centre of the Department of General and Visceral Surgery (ulmCARES), University Hospital Ulm, Albert-Einstein-Allee 23, 89081 Ulm, Germany Patrick Heger, Colette Dörr-Harim, André L. Mihaljevic (former address): Department of General, Visceral and Transplantation Surgery, University Hospital Heidelberg, Im Neuenheimer Feld 420, 69120 Heidelberg, Germany Thilo Hackert, Markus W. Büchler: Department of General, Visceral and Transplantation Surgery, University Hospital Heidelberg, Im Neuenheimer Feld 420, 69120 Heidelberg, Germany Markus K. Diener: Department of General and Visceral Surgery, University Hospital Freiburg, Hugstetter Straße 55, 79106 Freiburg, Germany Manuel Feißt, Christina Klose: Institute of Medical Biometry (IMBI), University of Heidelberg, Im Neuenheimer Feld 130.3, 69120 Heidelberg, Germany Friedhelm Möhlenbrock: Arbeitskreis der Pankreatektomierten e.V., Thomas-Mann-Straße 40, 53111 Bonn, Germany
Name and contact information for the trial sponsor {5b}	André L. Mihaljevic: Department of General and Visceral Surgery, University Hospital Ulm, Albert-Einstein-Allee 23, 89081 Ulm, Germany Phone: +49 (0) 731-500-53502 Fax: +49 (0) 731-500-53503 Email: andre.mihaljevic@uniklinik-ulm.de
Role of sponsor {5c}	The funding body (German Ministry of Research and Education, Bundesministerium für Bildung und Forschung BMBF, Funding number 01KG2129) has no role in the study design, collection, management, analysis, and interpretation of the data; writing of the report; and the decision to submit the report for publication. Furthermore, it has no ultimate authority over any of these activities.

## Introduction

### Background and rationale {6a}

Pancreatic ductal adenocarcinoma (PDAC) is the fourth most frequent cause of cancer-related death in the Western world, and its incidence is rising [1, 2]. Survival differs widely depending on the tumour stage and surgical resection via pancreatoduodenectomy (PD) in case of tumours of the pancreatic head is one of the mainstays in the treatment of PDAC and the only option for curative treatment [3–5]. The rise of more efficient chemotherapeutic regimes like FOLFIRINOX or Gem/nabPac in recent years, has led to an increased use of neoadjuvant treatments raising the hope that more patients will qualify for potential curative resection in the future [6].

However, local recurrence (LR) is frequent following surgical resection for PDAC, and prognosis is significantly worse for patients experiencing LR compared to non-LR [7].

Several independent risk factors predict LR, one of which is the status of the pathological resection margin. The R0 resection rate has been proposed as a quality indicator for pancreatic surgery [8, 9] and has shown to be an important prognostic parameter not only for local recurrence but can also improve overall survival (OS) as well as disease-free survival (DFS) in PDAC patients [10].

Owing to the perineural growth pattern of PDAC, R1 resections occur most frequently along the soft tissue margins towards the celiac and superior mesenteric artery (i.e. medial margin) [11, 12]. Therefore, by applying more radical dissection techniques along the superior mesenteric artery (SMA) and the celiac artery (CA), marginal clearance can be improved.

Concerning the radicality around the SMA, Inoue et al. have proposed a classification system including 3 levels of dissection around the SMA [13]. A recently proposed surgical technique goes beyond level 3 SMA dissection by additionally resecting all soft tissue between the CA, the SMA and the mesenterico-portal axis (MPA) (the so-called triangle) [14, 15]. This operation leads to clearance of all soft and lymphatic tissue which drains the pancreatic head and seems a promising approach as LR following PDAC resection frequently occurs in the “triangle” region between CA, SMA and MPA [16–18]. In addition, the R0 status along the posterior and medial margins has been associated with improved local recurrence and DFS in a recent study [19]. Consequently, the TRIANGLE operation has the potential to improve LR and DFS following PD surgery for pancreatic head cancer [10, 13]. However, high-quality data in terms of a multicentre randomised controlled trial are lacking so far. Therefore, the TRIANGLE trial aims to evaluate whether the “TRIANGLE” operation increases DFS in patients with carcinomas of the pancreatic head.

### Objectives {7}

The TRIANGLE trial aims to evaluate whether a more radical dissection along the SMA and removal of all lymphatic and soft tissue between the CA, the MPA and the SMA (the so-called triangle) during PD in comparison with conventional PD increases DFS in patients with carcinomas of the pancreatic head.

### Trial design {8}

The TRIANGLE trial is a multicentre confirmatory randomised controlled patient and outcome assessor blinded superiority trial with two parallel study groups.

## Methods: participants, interventions and outcomes

### Study setting {9}

The trial will be carried out in at least seven pancreatic cancer centres. Most of the centres are part of the Clinical Trial Network of the German Society of Surgery (CHIR-Net; www.chir-net.de). A list of the trial sites can be found in Additional file 1.

### Eligibility criteria {10}

The following are the preoperative inclusion criteria for patients:

- Patients with suspected or histologically verified resectable, borderline or locally advanced pancreatic cancer of the pancreatic head (i.e. pancreatic ductal adenocarcinoma, intraductal papillary mucinous neoplasm (IPMN)-carcinoma or periampullary cancer of the pancreatobiliary-type)
- Patients scheduled for elective partial pancreatoduodenectomy (irrespective of neoadjuvant therapy)
- Assumed resectability in accordance with the surgical protocol for experimental and control intervention as judged by the treating surgeon
- Ability of the subject to understand character and individual consequences of the clinical trial
- Written informed consent
- Age ≥ 18 years

The following are the intraoperative inclusion criteria for patients (prior to randomisation):

- No distant metastases
- No paraaortic lymph node metastases
- Intraoperative confirmation that the patient can be operated on according to both surgical methods (experimental or control group)

The following are the exclusion criteria for patients:

- Participation in another interventional trial with the interference of intervention and outcome of this trial
- American Society of Anesthesiologists (ASA) grade > 3
- Distant metastatic disease

### Eligibility criteria for trial centres

All participating trial sites will be high-volume centres with broad expertise in pancreatic surgery and have the necessary expertise, equipment and personnel to perform this trial.

### Who will take informed consent? {26a}

All patients scheduled for PD will be screened preoperatively with regard to the inclusion and exclusion criteria. An authorised investigator will inform the patient, orally and written, about the aims of the trial, the possible risks, the procedures, the possible hazards to which he/she will be exposed and the mechanism of treatment allocation (randomisation). The written informed consent form will be signed and personally dated by the patient according to the ICH guidelines in Good Clinical Practice.

### Additional consent provisions for collection and use of participant data and biological specimens {26b}

No additional biological samples will be collected during the trial.

### Interventions

#### Explanation for the choice of comparators {6b}

As outlined in the introduction, radical extended PD including the removal of the tissue in the TRIANGLE area could improve the R0 resection rate and thereby the survival of patients suffering from pancreatic cancer. So far, high-quality data in terms of a multicentre randomised controlled trial on the TRIANGLE intervention are lacking. Therefore, a trial evaluating the effect of the TRIANGLE intervention compared to standard PD according to the current guidelines is needed.

#### Intervention description {11a}

##### Exploration phase (experimental and control group)

The experimental intervention is limited to the resection phase. Thus, both groups (experimental and control) start with the same exploratory phase. After safe access to the abdominal cavity, the exploration phase should comprise the following steps, which can be performed in any order (at the discretion of the surgeon):

1. Exclusion of hepatic metastases and peritoneal carcinomatosis. If distant metastases are present, the patient must not be randomised.
2. Dissection of the gastrocolic ligament to open the lesser sack and define tumour extent along the pancreatic gland and the stomach. No transection is performed at this time.
3. Any type of artery-first approach may be performed to explore potential tumour infiltration [20]. If resection of the tumour is not possible based on intraoperative findings, the patient must not be randomised.
4. A Kocher manoeuvre is performed to expose the para-aortic lymph nodes (LNs) and the root of the SMA.
  - If a suspicious para-aortic LN is detected, it should be resected and sent for frozen section.
  - In case the frozen section procedure reveals tumour infiltration, no randomisation should be performed as available data shows that the prognosis of patients with positive para-aortic LNs is poor (ISGPS guidelines [21]).
5. After the exclusion of distant and paraaortic LN metastases and intraoperative confirmation that the patient can be operated according to both experimental or control groups, randomisation will be performed.

##### Resection phase—experimental group (TRIANGLE operation)

The experimental intervention consists of two parts:

A Dissection of the SMA according to level 3 described by Inoue et al. [13]. This step includes a dissection of the nerve plexus around the superior mesenteric artery (plSMA) from at least 5 to 11 o’clock (180°). A wider resection (≥ 180°) up to a circular (360°) resection of the lymph plexus and plSMA is allowed and at the discretion of the surgeon. Performing this step should result in a circular (360°) dissection of the superior mesenteric vein (SMV).
B Complete dissection of the soft tissue in the “triangle” between CA, SMA and MPA [14].

##### Resection phase—control group

Patients randomised to the control group will receive standard PD with dissection of the SMA according to Inoue level 1 or 2 and standard lymphadenectomy according to the German S3 guidelines [8] but no TRIANGLE operation. This constitutes the current standard of care.

Deviations from the described control intervention with venous or arterial reconstruction or extended pancreatectomy in case of advanced tumour infiltration are possible as long as there is no radicality necessary according to Inoue level 3 along the SMA or removal of soft tissue in the triangle area.

##### Resection phase—experimental and control groups

The further key steps of the resection phase in the experimental as well as the control group should include lymphadenectomy in the hepatoduodenal ligament as described in the German S3 guidelines [8]. Furthermore, the transected end of the bile duct and the transection of the pancreatic body should be sent for frozen section to rule out microscopic tumour infiltration. In case of tumour infiltration in the pancreatic body, further resection of the pancreatic body is indicated. Transection of the postpyloric duodenum or the prepyloric stomach should be performed as oncologically necessary. Therefore, pylorus-preserving, pylorus-resecting or classical Whipple procedures or any variants are allowed in the trial. If extended pancreatic resections (e.g. total pancreatectomy) or resection of neighbouring organs are necessary, patients remain in the trial. Details should be given during visit 2 (surgery). Any kind of venous or arterial resection and reconstruction can be performed as deemed necessary by the operating surgeon.

##### Reconstruction phase—experimental and control groups

The reconstruction phase is the same in both groups including pancreatojejunostomy or pancreaticogastrostomy, hepaticojejunostomy and a duodeno- or gastrojejunostomy according to local standards. Similarly, the placement of drains, abdominal wall and skin closure should be performed according to local standards. Details will be recorded during visit 2.

If reconstruction needs to be performed later in a second operation, this is possible and does not result in the exclusion of the patient from the trial.

### Criteria for discontinuing or modifying allocated interventions {11b}

After inclusion into the trial, patients still have to meet the intraoperative inclusion criteria to be finally randomised to one of the intervention groups. If during the exploration phase distant metastases or paraaortic lymph node metastases are found or it can not be confirmed that the patient can be operated on according to both surgical methods (experimental or control group), the patient has to be excluded intraoperatively and will be defined as an intraoperative drop-out. Furthermore, if, in the investigator’s opinion, continuation of the trial intervention would be detrimental to the subject’s well-being, the investigator may stop the trial intervention for this patient. In this case, the reason for the individual premature trial termination must be recorded in the electronic case report form (eCRF) and in the patient’s medical records.

### Strategies to improve adherence to interventions {11c}

As the interventions of the TRIANGLE trial (experimental and control groups) are surgical interventions, strategies to improve adherence to the allocated intervention are not needed.

### Relevant concomitant care permitted or prohibited during the trial {11d}

Additional perioperative care of the patients will be performed according to the institutional standards. All additional medications and/or treatments are permitted during the trial when considered necessary by the treating physician.

### Provisions for post-trial care {30}

The post-trial care of the patients will be the regular tumour follow-up according to the current guidelines. No compensation will be provided.

### Outcomes {12}

#### Primary endpoint

The primary endpoint of the TRIANGLE trial will be the DFS after resection, defined “as the time from randomisation until disease recurrence or death from any cause” [22]. Disease recurrence can be a local recurrence or distant metastases. Given the overall dismal prognosis of PDAC, the high rate of recurrence as well as the objective of the trial, i.e. to reduce recurrence rates and improve OS, DFS seems the logical and most relevant endpoint for the TRIANGLE trial. It is a frequent endpoint in cancer trials in the adjuvant setting after definitive surgery, and it is widely accepted by regulatory authorities worldwide [22, 23] and has been used in previous randomised controlled trials (RCTs), thus allowing the comparison of data between trials [16, 24, 25]. Furthermore, it is based on objective and quantitative assessments. DFS is patient-relevant as it includes all-cause mortality and recurrence. In order to avoid detection bias, follow-up will be standardised for both groups and include clinical outpatient visits, contrast-enhanced CT scan and tumour markers every 6 months for 3 years.

#### Primary estimand

In the recently released addendum to the ICH E9 guideline (final version), the estimand framework is recommended as clear and transparent definition of “what needs to be estimated to address a specific scientific question of interest”. Such an estimand should be defined through the treatment condition of interest, the population of interest, variable of interest, specification of how intercurrent events are handled, and summary measure. The specification of how intercurrent events are handled is referred to as intervention effect in the following. This way a more precise definition of the treatment effect of interest in relation to the trial objective(s) is enabled. Based on such an estimand, adequate methods to estimate this estimand can be chosen. In the following, the primary estimand (see Table 1) corresponding to the primary objective is described.

**Table 1:** Overview of the primary estimand

**Analysis**	**Treatment**	**Population**	**Variable**	**Intervention effect (strategy)**	**Summary measure**
Primary	Specified in Intervention description (11a)	Defined by the in- and exclusion criteria	DFS	Death: composite Incomplete observation due to loss to follow-up or early drop-out: hypothetical Others: treatment policy	Hazard ratio (Cox proportional hazards regression model)

Treatment: The treatments patients will receive in the experimental group or in the control group of the trial are specified as mentioned above.

Population: The targeted population is defined through the in- and exclusion criteria.

Variable: The variable is disease-free survival after resection, defined as the time from randomisation until disease recurrence (local recurrence or distant metastases) or death from any cause.

Intervention effect: Possible intercurrent events and the strategies to handle them are as follows: Death, as an intercurrent event occurring after randomisation, is handled by inclusion into the definition of the primary endpoint which reflects a composite strategy. Patients with incomplete observation time due to loss to follow-up or early drop-out will be censored at the last observation, which reflects a hypothetical strategy. Besides these events, other post-randomisation events (e.g. intervention not as randomised, discontinuation of chemotherapy) will not be considered, thus reflecting a treatment policy approach, which means that the effect of randomised treatment is estimated irrespectively of other post-randomisation events not captured in the primary endpoint definition. This corresponds to the intention-to-treat (ITT) principle.

Summary measure: The summary measure is the adjusted hazard ratio.

#### Secondary endpoints

Secondary endpoints of the TRIANGLE trial are as follows:

1. Rate of the following:
  - Microscopically complete margin clearance (> 0.1 cm margin clearance, R0(CRM-))
  - Microscopic margin clearance ≤ 0.1 cm (R0(CRM+))
  - Microscopic margin involvement (R1) resections according to the 8th edition of the UICC TNM classification
2. Rate of the following PD-associated postoperative complications within 90 days after the index operation:
  - Postoperative pancreatic fistula (POPF) as defined by the International Study Group of Pancreatic Surgery (ISGPS) [26]
  - Postpancreatectomy haemorrhage (PPH) as defined by the ISGPS [27]
  - Delayed gastric emptying (DGE) as defined by the ISGPS [28]
  - Bile leakage as defined by the International Study Group of Liver Surgery (ISGLS) [29]
  - Lymphatic fistula as defined by the ISGPS [30]
  - Diarrhoea as graded by the Common Terminology Criteria for Adverse Events (CTCAE) version 5.0 [31]
3. All other postoperative complications graded according to the Dindo-Clavien classification [32] within 90 days. This endpoint complements the PD-associated complications evaluated via the above-mentioned ISGPS endpoints, to assess all remaining postoperative complications to establish a risk-benefit assessment of the two interventions.
4. Overall survival within the study period.
5. Local recurrence within the study period.
6. Quality of life (QoL) according to the European Organisation for Research and Treatment of Cancer (EORTC) QLQ-C30 and PAN26 at discharge and during every follow-up visit compared to baseline.
7. Quality of recovery (QoR) according to the QoR-15 questionnaire on postoperative day 5 compared to baseline.
8. Length of primary hospital stay in days from the day of index operation to the day of discharge.
9. Serious adverse events (SAEs) in both groups .

### Participant timeline {13}

**Fig. 1:**
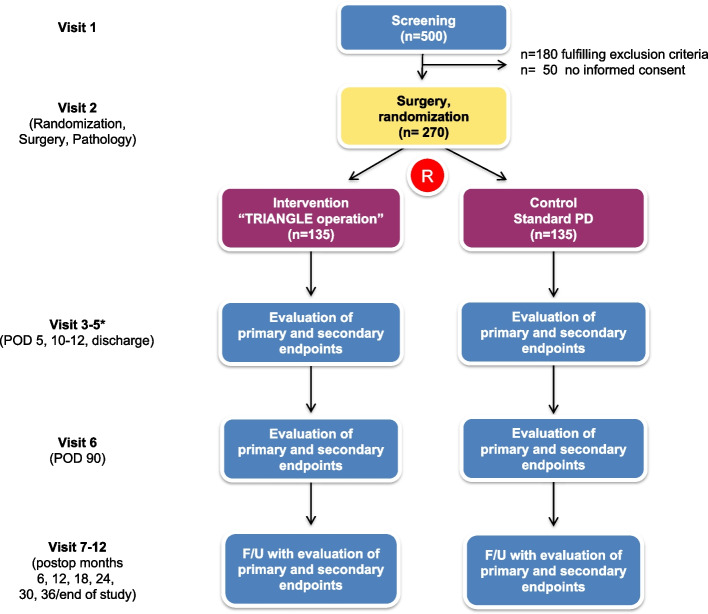
Flow chart of the TRIANGLE trial. F/U, follow-up; POD, postoperative day; PD, partial pancreaticoduodenectomy; R, randomisation. *Respective visits are skipped if the patient has been discharged before

Patients scheduled for elective PD will be screened preoperatively (visit 1). After the patient has given informed consent, the inclusion and exclusion criteria are assessed during the screening visit. Patients fulfilling all the inclusion criteria and none of the exclusion criteria and who consent to take part in the trial are randomised during surgery (visit 2; surgery and randomisation) after the surgeon has obtained certainty that both interventions can be performed in the patient. The results of the pathologic work-up (secondary endpoint) will also be recorded in the eCRF section of visit 2. Patients are planned for follow-up visits on postoperative days 5, 10–12 and 90 (visits 3, 4 and 6) for the evaluation of primary and secondary endpoints. Visit 5 will be performed at discharge. If discharge occurs before visit 3 or 4, the respective postoperative visits (3 or 4) can be omitted. In addition, 6, 12, 18, 24, 30 and 36 months (visits 7–12) after surgery, patients are planned for follow-up visits to evaluate primary and secondary outcome parameters (Fig. 1 and Table 2).

**Table 2:** Trial visits and documented parameters

**Visit**	**1**	**2**	**3–4**^a^	**5**	**6**	**7–12**
**Time point**	Screening	Day of surgery, pathology	POD 5 POD 10–12	Day of discharge	POD 90	POM 6, 12, 18, 24, 30, 36 (or pre-mature trial termination)
**Type of visit**	Inpatient	Inpatient	Inpatient	Inpatient	Outpatient (inpatient) (telephone)	Outpatient (telephone)
Written informed consent	**X**					
Preoperative inclusion/exclusion criteria	**X**					
Intraoperative inclusion criteria		**X**				
Demographics and baseline data	**X**					
Medical history	**X**					
Surgery data		**X**				
Randomisation		**X**				
*Primary endpoint*						
Disease-free survival			**X**	**X**	**X**	**X**
*Secondary endpoints*						
Pathologic data		**X**				
Postoperative morbidity			**X**	**X**	**X**	
Length of hospital stay				**X**		
Overall survival			**X**	**X**	**X**	**X**
Local recurrence			**X**	**X**	**X**	**X**
*Patient-reported outcomes (PRO)*						
Quality of recovery^c^	**X**		**(X)**^d^			
Quality of life assessment^b^	**X**			**X**		**X**
*Safety*						
SAE		**X**	**X**	**X**	**X**	**X**

*POD* Postoperative day, *POM* Postoperative month, *PRO* Patient-reported outcome, *SAE* Serious adverse events

^a^Skip respective visits if the patient has been discharged prior to visit

^b^According to EORTC QLQ-C30 and -PAN26

^c^According to the QoR-15

^d^Only on POD 5

### Sample size {14}

The sample size calculation is based on the primary endpoint disease-free survival. The assumptions are based on a median survival time of 14 months in the control group and 21 months in the treatment group resulting in a corresponding hazard ratio of 0.667 assuming exponentially distributed survival times [7, 16, 33]. To detect the assumed difference using a log-rank test at a significance level of 5% (two-sided) with a power of 80%, a total number of 192 events (i.e. recurrence or deaths) are required for the entire trial. It is expected that applying the Cox proportional hazards model, as described below, will lead to an increase in power. With an accrual period of 30 months and a follow-up period of at least 36 months, and assuming an exponentially distributed drop-out rate of 20% (at 66 months), *n* = 270 (135 per group) patients are needed (based on the approximation formula by Schoenfeld). The sample size calculation was done using ADDPLAN 6.1.1.

### Recruitment {15}

In order to recruit the necessary number of patients, at least seven pancreatic cancer centres will participate in this trial. All participating trial sites will be high-volume centres with broad expertise in pancreatic surgery and have the necessary expertise, equipment and personnel to perform this trial. Most of the centres are part of the CHIR-Net.

### Assignment of interventions: allocation

#### Sequence generation {16a}

To ensure an equal distribution of patient characteristics and confounders between the two groups, a randomisation tool will be used. Randomisation will be performed at the patient level with a centralised online randomisation system (www.randomizer.at). The online randomisation procedure provides information regarding the group allocation and a randomisation number. The randomisation sequence is computer-generated and will be stratified by centre and presence/absence of neoadjuvant treatment. Block randomisation will be performed. The randomisation procedure will be performed intraoperatively (after surgical exploration) after the fulfilment of all intraoperative inclusion criteria.

#### Concealment mechanism {16b}

As randomisation in the TRIANGLE trial depends on the intraoperative inclusion criteria and the findings during the exploratory phase of surgery, the allocation to the assigned group will be performed intraoperatively after fulfilling the inclusion criteria. Allocation concealment is therefore not necessary, as the assigned intervention will be performed immediately after randomisation.

#### Implementation {16c}

The randomisation will be performed intraoperatively by a member of the study team after fulfilling all of the intraoperative inclusion criteria by using the online randomisation tool. Names of the randomising study team member as well as the team of surgeons will be documented. They will not be involved in the outcome assessment.

### Assignment of interventions: blinding

#### Who will be blinded {17a}

Patients and outcome assessors will be blinded to the intervention in order to guarantee an unbiased assessment of the primary and secondary endpoints. The outcome assessors will be neither part of the surgical team that performs the trial intervention nor have access to the randomisation tool. All patient-reported outcomes (PROs) will be assessed using validated measures. Moreover, the discharge letter will contain no information regarding group allocation.

#### Procedure for unblinding if needed {17b}

If unblinding is necessary due to medical conditions, it can be performed by the treating physicians or one of the unblinded study team members. Unblinding will be documented and reported to the steering group of the trial.

### Data collection and management

#### Plans for assessment and collection of outcomes {18a}

All of the included trial sites will be high-volume centres with broad expertise in pancreatic surgery as well as the conduction of clinical trials. Most of the centres are part of the CHIR-Net, which has successfully performed trials with similar indications and recruitment rates in the past [34–36].

To further enhance data quality and to minimise detection bias, validated measures and classifications will be used where possible (see the “Outcomes {12}” section). Additionally, the primary outcome (DFS) is an objective measure defined “as the time from randomisation until disease recurrence or death from any cause” according to international guidelines [22].

#### Plans to promote participant retention and complete follow-up {18b}

As outlined above, all of the included trial sites will be experienced in the conduction of randomised trials with similar indications and follow-up procedures. Furthermore, pancreatic cancer patients are in need of an oncological follow-up that usually is performed in high-volume centres. If follow-up is not performed in the centres, visits after discharge of the patients can also be done by telephone including a collection of the necessary data.

#### Data management {19}

An eCRF will be used for data collection. The study data will be collected using REDCap [37] (Research Electronic Data Capture), a secure, web-based data capture application hosted at the IMBI. To assure a safe and secure environment for the data acquired, data transmission is encrypted with secure socket layer (SSL) technology. The database server is located in a secure data centre and is protected by a firewall. The system provides an infrastructure to support user roles and rights. Only authorised users are able to enter or edit data; the access is restricted to data of the patients in the respective centre. All changes to data are logged with a computerised timestamp in an audit trial. All clinical data will be pseudonymised. Backups are conducted regularly.

All protocol-required information collected during the trial must be entered by the investigator or designated representative in the eCRF. For health-related quality of life and patient-reported outcome data, patients may directly enter the data in the eCRF. Alternatively, paper-based reported outcome questionnaires must be entered by the investigator or designated representative in the eCRF. The investigator or designated representative should complete the eCRF forms as soon as possible after information is collected, preferably on the same day that a trial subject is seen for an examination, treatment, or any other trial procedure. Any outstanding entries must be completed immediately after the final examination. An explanation should be given for all missing data. The completed eCRF must be reviewed and signed by the local investigator or by a designated sub-investigator.

To guarantee high data quality, data validation rules will be defined in a data validation plan. Completeness, validity and plausibility of data will be checked at the time of data entry (edit checks) and using validating programs, which will generate queries. The investigator or the designated representatives are obliged to clarify or explain the edit checks and queries. If no further corrections are to be made in the database, eCRF data will be locked.

All data collected will be integrated into a statistical analysis system. During study conduct, database access will be granted to the data manager only. After database closure, access rights will be granted to the biometricians as well. The data will be managed and analysed in accordance with the appropriate standard operating procedures (SOPs) valid in the IMBI Heidelberg that guarantee an efficient conduct complying with Good Clinical Practice (GCP). Photo files from the surgery are collected at each local site and are stored according to the applicable local, national and international regulations.

#### Confidentiality {27}

Patients will be informed as to the strict confidentiality of their data, but that their medical records may be reviewed for trial purposes by authorised individuals (trial monitor) other than their treating physician. It is the responsibility of the investigator to maintain patients’ confidentiality. During the trial, patients will be identified solely by means of their individual identification codes. Trial-specific documents will be stored in accordance with local data protection law/ICH-GCP Guidelines and will be handled in the strictest confidence. For the protection of these data, organisational procedures are implemented to prevent the distribution of data to unauthorised persons. The patients’ data will be transferred in a pseudonymised form from the trial centre to cooperating partners (coordinating investigator, data management). Names and all confidential data of participating patients will be handled in line with the obligations of medical secrecy, the European General Data Protection Regulation (Datenschutzgrundverordnung, DSGVO), the Federal Data Protection Act (Bundesdatenschutzgesetz) and the state Data Protection Act (Landesdatenschutzgesetz). Participating patients’ data will be documented in the eCRF only in pseudonymised form. Decoding of the pseudonymised data is only permitted in justified cases. Third parties have no access to original documents. After completion of the trial, data collected during the study will be kept on file for 10 years. It is guaranteed that the data protection provisions are followed. The sponsor provides data protection management and an information security management system. The Clinical Trial Centres are contractually obliged to comply with the DSGVO and other data protection regulations.

#### Plans for collection, laboratory evaluation and storage of biological specimens for genetic or molecular analysis in this trial/future use {33}

There are no plans for the collection, laboratory evaluation and storage of biological specimens for genetic or molecular analysis in the TRIANGLE trial.

## Statistical methods

### Statistical methods for primary and secondary outcomes {20a}

The primary outcome disease-free survival is compared between the groups using a Cox proportional hazards model adjusted for the covariates treatment group, age, neoadjuvant treatment, adjuvant treatment as fixed effects and centre as a random effect. It can be expected that applying the Cox proportional hazards model will lead to an increase in power in comparison with a log-rank test. Confounding by other less important prognostic and predictive factors can be assumed to be controlled by the randomised trial design. The significance level is set to 5% (two-sided). The confirmatory analysis is conducted based on the full analysis set (FAS) according to the ITT principle, i.e. all randomised patients are included and analysed in the treatment group as randomised which reflects a treatment policy approach (according to the ICH E9 (R1) addendum). Death, as an intercurrent event occurring after randomisation, is handled by inclusion into the definition of the primary endpoint which reflects a composite strategy.

All secondary outcomes will be evaluated descriptively, and descriptive *p*-values for the corresponding effects will be reported along with 95% confidence intervals. Continuous variables will be described using a number of missing values, non-missing values, mean, standard deviation, median, Q1, Q3, minimum and maximum. For binary or categorical variables, absolute and relative frequencies will be provided. Thereby, binary and categorical variables will be compared by the chi-squared tests, and continuous variables will be compared with *t*-tests. OS will be analysed analogously to the primary endpoint with a Cox proportional hazards model. QoL will be analysed using linear mixed models with baseline score, age, treatment group and the stratification variables as covariates; centre and time point will be included as random effects. Furthermore, the treatment effect for secondary endpoints will be assessed descriptively within several subgroups to identify potential prognostic and predictive factors. Analyses of secondary endpoints will be based on the ITT population. No imputation of missing values will be conducted in the analyses of secondary endpoints.

Baseline characteristics will be analysed descriptively with appropriate statistical measures. Further details of the analysis will be specified in the statistical analysis plan (SAP) which will be finalised before database closure. All calculations will be done using SAS version 9.4 or higher.

### Interim analyses {21b}

There are no interim analyses planned.

### Methods for additional analyses (e.g. subgroup analyses) {20b}

As a sensitivity analysis in the meaning of the ICH E9 addendum, the primary estimand will also be analysed by the unadjusted Cox proportional hazards model. As supplementary analyses, the primary endpoint will also be evaluated based on the per-protocol (PP) population including only those patients without major protocol violations and based on the as-treated set the same way as in the confirmatory analysis. Furthermore, the treatment effect will be assessed descriptively within several subgroups to identify potential prognostic and predictive factors. In addition, further covariables are included and analysed in the primary analysis model in order to identify further possible influencing factors.

For safety analysis, all SAEs will be analysed via descriptive statistical methods. The safety analysis includes calculation of frequencies and rates of complications and serious adverse events together with corresponding 95% confidence intervals. For comparisons of frequencies between the groups, the chi-square test will be used.

### Methods in analysis to handle protocol non-adherence and any statistical methods to handle missing data {20c}

The confirmatory analysis of the primary endpoint will be conducted according to the ITT principle which reflects a treatment policy approach according to the ICH E9 (R1) addendum. Patients with incomplete observation time due to loss to follow-up or early drop-out will be censored at the last observation (hypothetical strategy). Further post-randomisation events will be ignored. This also reflects a treatment policy approach. Because of the use of the Cox approach, no missing values in the primary endpoint will occur. Furthermore, no missing values in the covariates of the Cox model are expected.

### Plans to give access to the full protocol, participant-level data and statistical code {31c}

The full protocol will be accessible with this publication. The participant-level dataset will be available anonymised after the publication of the final results of the study.

### Oversight and monitoring

#### Composition of the coordinating centre and trial steering committee {5d}

The trial will have a steering committee consisting of the trial statistician and three clinical experts. The steering committee will supervise the conduct of the trial and will issue recommendations for early termination, modifications or continuation of the trial, if necessary.

#### Composition of the data monitoring committee, its role and reporting structure {21a}

Two independent experts (surgeon and biostatistician) and one patient representative from the “Arbeitskreis der Pankreatektomierten e.V.” (AdP) will constitute a data safety monitoring board (DSMB). DSMB members will meet on a regular basis to monitor and supervise the progress of the trial. For these meetings, they will receive a written DSMB report within the active phase of the trial (recruitment) at least once a year and will advise on the continuation, modification or termination of the trial. This safety report will include numbers on the current recruitment status as well as major complications that happened. Further details can be found in the separate DSMB charter. The coordinating investigator and/or the steering committee may call upon the DSMB in case a safety issue arises during the course of the trial.

#### Adverse event reporting and harms {22}

An SAE within the TRIANGLE trial is any adverse event (AE) (= any untoward medical event) occurring at any time during the period of observation, which results in death, is immediately life-threatening or requires or prolongs hospitalisation for a complication associated with the index surgery.

Since the interventions of the TRIANGLE trial are medical routine and the trial is conducted according to the medical association’s professional code (Berufsordnung der Bundesärztekammer) §15, there is no need for a specific SAE management. Anyhow, all documented SAEs will be summarised and considered periodically. All SAEs need to be recorded in the SAE form. Reporting has to be performed within 10 days after the SAE has become known. SAEs need to be documented from randomisation (visit 2) to the end of the trial (visit 12).

#### Frequency and plans for auditing trial conduct {23}

During the clinical trial, quality control and quality assurance will be ensured via monitoring. Clinical monitoring will be performed regularly by the independent monitoring department of the “Zentrum für Klinische Studien” (ZKS) Ulm according to its standard operating procedures. The objectives of the monitoring procedures are to ensure that the subject’s safety and rights of a clinical trial participant are respected; that accurate, valid, and complete data are collected; and that the clinical trial is conducted in accordance with the protocol, the principles of ICH and applicable regulatory and local requirements. Monitors therefore must be allowed to access patient’s hospital records and other source documentation upon request. A risk-based monitoring strategy will be conducted based on patient safety, patient rights, protocol adherence and data validity and a study-specific pre-defined monitoring plan.

The frequency of monitoring visits will be determined depending on recruitment numbers and individual performance of each centre based on feedback from project and data management and according to the risk-based monitoring approach.

#### Plans for communicating important protocol amendments to relevant parties (e.g. trial participants, ethical committees) {25}

The ethics committee will be informed of any amendment to the protocol and asked if formal approval is necessary or if further revision of trial documents should be performed. All amendments to the protocol will be communicated to the participating centres immediately.

#### Dissemination plans {31a}

The results of the TRIANGLE trial will be reported according to the recommendations of the CONSORT statement and publication in international open-access peer-reviewed journals is intended. The final report of the trial will be reviewed by all trial sites. Furthermore, the results will be communicated to appropriate patient organisations and will be presented at international conferences.

## Discussion

LR is one of the most frequent reasons limiting DFS in curatively treated PDAC patients by multimodal therapy including surgery. A recent study analysing recurrence patterns in PDAC revealed that in 20.8% of the patients, LR occurred alone and another 27.8% of the patients exhibited PDAC recurrence at multiple sites, almost always including LR as a primary recurrence site [7]. As the R status showed to be an independent determinant of recurrence-free survival [10], it seems like treatment strategies to improve the R0 resection rate could reduce the rate of LR and therefore improve DFS.

The TRIANGLE trial will evaluate the effect of the recently proposed surgical technique in PDAC surgery that includes level 3 SMA dissection as described by Inoue et al. [13] and additionally the TRIANGLE intervention resecting all soft tissue between the CA, the SMA and the MPA [14]. Finally, all soft and lymphatic tissue which drains the pancreatic head will be resected. The rationale for this surgical approach stems from radiologic and imaging studies that confirm the lymphatic and perineural tumour invasion in the area between the CA, the SMA and the MPA [18]. This hypothesis is further supported by the recurrence patterns following PDAC resection, as LR frequently occurs in this area [16–18]. Additionally, a recent study has proposed the R0 status along the posterior and medial margins to be associated with improved local recurrence and DFS [19]. Finally, pathologic studies, which have applied standardised pathologic work-up and reporting, indicate that the medial resection margin (i.e. the soft tissue margins towards the CA and the SMA) is the most frequent site of microscopic tumour involvement [12, 38–40]. Margin clearance in turn has been shown to be an important prognostic parameter of survival as discussed above.

This potential oncologic advantage of the TRIANGLE operation must be weighed against potential AEs and increased morbidity which might be associated with more radical medial margin clearance. In the study by Inoue et al., with a total of 82 patients, 12 out of 14 patients suffering from diarrhoea after surgery had level 3 SMA dissection [13]. However, the frequency of other complications was comparable [13]. This is in line with previous reports investigating extended interaortocaval lymphadenectomy in PDAC which showed an increase in morbidity [8, 21]. Similarly, extended PD with multivisceral resections showed a significant increase in perioperative morbidity [41]. However, it should be pointed out that although PD can be performed with perioperative mortality below 4% in specialised centres [42], morbidity rates generally remain high between 30 and 40% even for conventional PDs and even in high-volume centres [42]. More data concerning the safety of extended PD stems from our PancER trial and the already published data of a retrospective analysis of more than 100 patients treated by the TRIANLGE approach [15].

In preparation of the TRIANGLE trial, we have initiated a monocentric trial with a similar indication (PancER - Conventional partial pancreatoduodenectomy versus an uncinate first, extended partial pancreatoduodenectomy approach for the resection of pancreatic head ductal adenocarcinoma, DRKS00013552). The PancER trial was a monocentric randomised controlled trial with the experimental intervention of an extended PD including only Inoue level 3 dissection along the SMA. No clearance of tissue in the “triangle” (= soft tissue between the CA, the SMA and the mesenterico-portal axis) was explicitly demanded in the PancER trial. The control groups (standard PD) were the same in both trials. Importantly, the number of serious adverse events in both groups in the PancER trial was comparable in an interim safety analysis of the first 45 patients. Similarly, in the same interim safety analysis, the number of surgery-associated morbidity within 30 days was not significantly different between the groups (pancreatic fistula, delayed gastric emptying, chyle leak, haemorrhage), except diarrhoea, which occurred significantly more often in the interventional group (5.9% control group vs. 53.6% experimental group; *p* = 0.014). However, diarrhoea can frequently be successfully treated with antidiarrhoeal medication. These results confirmed the safety of the experimental trial procedure. The PancER trial was prematurely terminated on August 19, 2020, as recruitment of the trial was behind expectations and as the more promising TRIANGLE intervention was evolving. For the preparation of the TRIANGLE trial, the results of the PancER trial offered shortcomings in the recruitment process and served to improve this subsequent trial.

In addition to the PancER trial, the already published data of a retrospective comparison of patients treated by the TRIANGLE intervention to conventional PD served as preliminary data for the TRIANGLE trial. These results of 330 patients, of which 108 have been treated by the intervention of the TRIANGLE trial, have been published recently [15]. A reduced rate of R1 resections has been found in the TRIANGLE PD group compared to the conventional PD group without reaching significance (31.4% R1 in the TRIANGLE PD group compared to 42.9% R1 in the conventional PD group). Perioperative morbidity has been comparable between the groups with the exception of the rate of diarrhoea which was significantly higher after TRIANGLE PD (34.4% vs. 14.4%, *p* < 0.01).

In summary, the existing evidence offers indications that an extended PD as the TRIANGLE intervention could lead to improved R0 resection rates and therefore improved survival. However, high-quality data in terms of a multicentre randomised controlled trial are lacking so far. Similarly, high-quality data on the associated morbidity and the effects on patient-reported outcomes are missing, thus precluding an informed risk-benefit assessment of more radical approaches. These data will be collected in the planned TRIANGLE trial.

## Trial status

This manuscript was written according to the most current version of the study protocol (version 1.0, last updated on November 15, 2022). Recruitment of patients for the TRIANGLE trial will start in February 2023. The clinical phase of the trial (last patient out) is expected to be completed in July 2028.

## Supplementary Information

**Additional file 1.** List of study sites.

## Data Availability

After completion of the trial, the data obtained by the trial will be summarised and analysed according to this protocol and hereafter published in a peer-reviewed journal to be assessable by any healthcare professional, participant, or the public. Raw data or additional data can be requested on demand.

## Abbreviations

AdP: Arbeitskreis der Pankreatektomierten e.V.
AE: Adverse event
ASA: American Society of Anesthesiologists
BMBF: Bundesministerium für Bildung und Forschung
CA: Celiac artery
CTCAE: Common Terminology Criteria for Adverse Events
DFS: Disease-free survival
DGE: Delayed gastric emptying
DSMB: Data safety monitoring board
DSGVO: Datenschutzgrundverordnung
eCRF: Electronic case report form
EORTC: European Organisation for Research and Treatment of Cancer
FAS: Full analysis set
GCP: Good Clinical Practice
ICH: International Conference on Harmonization of Technical Requirements for Registration of Pharmaceuticals for Human Use
IMBI: Institute of Medical Biometry
IPMN: Intraductal papillary mucinous neoplasm
ISGLS: International Study Group of Liver Surgery
ISGPS: International Study Group of Pancreatic Surgery
ITT: Intention-to-treat
LN: Lymph node
LR: Local recurrence
MPA: Mesenterico-portal axis
OS: Overall survival
PD: Pancreatoduodenectomy
PDAC: Pancreatic ductal adenocarcinoma
pISMA: Nerve plexus around the superior mesenteric artery
POPF: Postoperative pancreatic fistula
PP: Per-protocol
PPH: Postpancreatectomy haemorrhage
PRO: Patient-reported outcome
QoL: Quality of life
QoR: Quality of recovery
RCT: Randomised controlled trial
SAE: Serious adverse event
SAP: Statistical analysis plan
SMA: Superior mesenteric artery
SMV: Superior mesenteric vein
SOP: Standard operating procedure
SSL: Secure socket layer
ZKS: Zentrum für Klinische Studien
