# Supplementary material for: Conventional partial pancreatoduodenectomy versus an extended pancreatoduodenectomy (triangle operation) for pancreatic head cancers—study protocol for the randomised controlled TRIANGLE trial
Source: Trials. 2023 May 30;24:363. doi: 10.1186/s13063-023-07337-6 (PMC10228091; doi:10.1186/s13063-023-07337-6)
Supplement: Supplementary file 1 — Additional file 1. List of study sites. [file 13063_2023_7337_MOESM1_ESM.docx]

| **Zentrum** | **Trial site** | **PI** |
| --- | --- | --- |
| Universitätsklinikum Bochum  Klinik für Allgemein- und Viszeralchirurgie | University Hospital Bochum  Department of General and Visceral Surgery | Prof. Dr. med. Waldemar Uhl |
| Universitätsklinikum Dresden  Klinik und Poliklinik für Viszeral-, Thorax- und Gefäßchirurgie | University Hospital Dresden  Department of Visceral, Thoracic and Vascular Surgery | Prof. Dr. med. Marius Distler |
| Universitätsklinikum Freiburg  Department Chirurgie  Klinik für Allgemein- und Viszeralchirurgie | University Hospital Freiburg  Department of General and Visceral Surgery | Prof. Dr. med Markus Diener |
| Universitätsklinikum Halle (Saale)  Klinik und Poliklinik für Viszerale, Gefäß- und Endokrine Chirurgie | University Hospital Halle (Saale)  Department of Visceral, Vascular and Endocrine Surgery | Prof. Dr. med. Ulrich Ronellenfitsch |
| Universitätsklinikum Hamburg-Eppendorf  Klinik für Allgemein-, Viszeral- und Thoraxchirurgie | University Hospital Hamburg-Eppendorf  Department of General, Visceral and Thoracic Surgery | PD Dr. med. Faik Uzunoglu |
| Universitätsklinikum Heidelberg  Klinik für Allgemein-, Viszeral, und Transplantationschirurgie | University Hospital Heidelberg  Department of General, Visceral and Transplantation Surgery | PD Dr. med. Rosa Klotz |
| Universitätsklinikum Ulm  Klinik für Allgemein- und Viszeralchirurgie | University Hospital Ulm  Department of General and Visceral Surgery | Prof. Dr. med. Andre Mihaljevic |
